# New approaches against the ancient pathogen *Mycobacterium tuberculosis*

**DOI:** 10.1016/j.ebiom.2021.103659

**Published:** 2021-10-27

**Authors:** EBioMedicine

*Mycobacterium tuberculosis* continues to be a leading cause of mortality worldwide, particularly in low-income and middle-income countries. According to WHO's Global Tuberculosis Report 2020, approximately 1·4 million worldwide die of the disease each year. 100 years after the BCG vaccine was first administered, it remains the only approved vaccine for tuberculosis, despite its variable efficacy (known to range between 0% and 80%), depending on exposure to mycobacteria and genetic and environmental differences. The rise of multidrug and extensively drug-resistant forms of *M tuberculosis,* coinfection with HIV, inadequate tests for latent tuberculosis, a slow developmental pipeline of drug and vaccine candidates, lengthy treatment, and the absence of rigorous biomarkers are the major obstacles to eradicating this disease. To reinvigorate the current approaches against tuberculosis, rapid and innovative strategies are urgently needed.

The complex interplay between the host immune system and *M tuberculosis* determines the fate of infection: latent disease, active disease, or, in very rare cases, rapid clearance. To help to divide latent and active tuberculosis along the clinical disease spectrum and to aid in diagnostic and therapeutic interventions, a recent review in *Clinical Microbiology Reviews* added subclinical and incipient tuberculosis to the list of disease states that can manifest in humans.

Biomarker-based or multiple-signature-based point-of-care tests to distinguish between tuberculous and non-tuberculous mycobacteria and between latent infection and active disease are urgently needed. A plethora of host and pathogen biomarkers including antibodies, cytokines, metabolic activity markers, and mycobacterial antigens have been identified. A systematic review published in *Nature Microbiology*, in 2019, reported that unfortunately only 4% of the total biomarkers assessed were of high quality (defined as meeting at least one WHO-approved targeted profile performance criterion). Metabolomic approaches that can be used on different types of samples, such as stool, serum, and faeces, are now being used to provide a quick, detailed analysis of a large number of samples.

Despite the presence of several well defined diagnostic assays (eg, Xpert), new point-of-care tests with increased sensitivity and specificity continue to be necessary. Distinguishing between *M tuberculosis* and *Mycobacterium avium* complex is important to aid timely isolation and provide the correct clinical interventions. Recently, Sarro and colleagues provided a glimmer of hope by reporting, in *EBioMedicine*, the development of a new multiplex assay capable of differentiating between the *M tuberculosis* and *M avium* complexes with a high analytical sensitivity threshold (5 colony-forming units per mL for *M tuberculosis* and *M avium* complexes, and 20 colony-forming units per mL for other non-tuberculous bacterium). The overall sensitivity of this assay in patients that responded to treatment was 83·3%, with a specificity of 96·6%, whereas the Xpert showed a sensitivity of 96·7% and specificity of 80·0% for the same patients, compared to sputum culture. The new test's better specificity but lower sensitivity compared with the Xpert and its ability to detect all *M avium* complex cases suggest that it could be used in settings where exposure to environmental mycobacteria is high.

Artificial intelligence has become a useful tool against tuberculosis, especially in high-burden countries where quick image triage is necessary and radiologist availability is scarce. A study recently published in *The Lancet Digital Health* evaluated five artificial intelligence algorithms in a large dataset consisting of 23 954 individuals, and showed that all the algorithms outperformed experienced human readers and reduced the need for Xpert tests by 50%, while still maintaining a sensitivity of more than 90%. These findings were not, however, without caveats: the tests performed worse for patients older than 60 years with a history of tuberculosis, probably because of a weakened immune response and residual pathogen in these individuals.

Machine learning can also be used to predict the clinical outcomes in tuberculosis and accelerate automatic identification of imaging biomarkers and validation of existing biomarkers. Tavolara and colleagues published two papers in *EBioMedicine in 2020* and 2021 that used Diversity Outbred mice to show that machine learning could identify super-susceptibility to tuberculosis infection from haematoxylin and eosin-stained lung tissue sections, using only clinical outcomes. In addition, their 2021 study showed that gene expression values (established from microarrays) from haematoxylin and eosin-stained whole-slide images could be used as an intermediate dataset to identify fulminant-like pulmonary tuberculosis. Gene expression predictions were shown to have a sensitivity of 0·88 and a specificity of 0·95 to identify super-susceptible mice (n=77), and a sensitivity of 0·88 and specificity of 0·93 for an external set of mice (n=33). Murine tuberculosis could be argued not to mimic human tuberculosis, and the time and resources required for machine learning might hamper the clinical utility of this technique. However, these studies allow for a proof of principle and should encourage further research in this field.

The microbiome might also have a role in tuberculosis pathogenesis, treatment, clinical outcomes, and post-treatment sequalae, and could be modulated to combat tuberculosis. A cross-sectional study by Naidoo and colleagues in *EBioMedicine*, in 2021, sheds some light on this hypothesis. This study looked at pre-treatment presumptive tuberculosis patients and negative symptomatic controls and contacts of both patients and controls without tuberculosis. Stool, sputum, and oral wash samples from the patients with presumed tuberculosis were enriched with anaerobes (namely *Paludibacter* spp*, Lautropia* spp, *Erysipelotrichaceae* spp*, Blautia* spp, and *Anaerostipesin* spp), and just the stool samples were enriched in microbial genes that were part of the amino acid and carbohydrate metabolic pathways. These anaerobes were negatively or positively correlated with enriched death receptor, eukaryotic initiation factor 2, interferon, and Nur77 (NR4A1) signalling and with inflammasome pathways, which were associated with disease severity, allowing for the potential targeting of these pathways in patients who are most affected.

There are currently 17 drugs and 12 vaccine candidates in the tuberculosis research pipeline, but progress in getting effective therapeutics to the clinic has been slow. A recent 30-year follow-up of a population-based, double-blind, randomised, placebo-controlled trial in Malawi published in *The Lancet Infectious Diseases* found little evidence of any beneficial effect of BCG revaccination on all-cause mortality. The positive response seen for the M72/AS01_E_ vaccine published in the *New England Journal of Medicine* has provided a much needed impetus for the vaccine pipeline.

The Stop TB partnership is a unique international body with the capacity to align actors from all over the world in the fight against tuberculosis. Achieving the aims set out by this partnership will require investment and political goodwill from leaders, increased drug adherence and seeking of medical health provision from patients, and commitment from health-care providers. Most importantly, development of new and innovative approaches that are tested in large, well validated cohorts comprising active, latent, incipient, and subclinical tuberculosis patients are needed. These advances require a deep understanding of the mechanisms used by *M tuberculosis* to invade and establish disease in humans. *EBioMedicine,* as a leading translational research platform, welcomes all research that can support efforts to probe these mechanisms and make the goals set out by the partnership a reality.

